# Andaliman (*Zanthoxylum acanthopodium* DC.) fruit ethanolic extract exerts attenuative effect on hyperglycemia, sensory and motoric function’s disorders in alloxan-induced diabetic mice

**DOI:** 10.5455/javar.2023.j716

**Published:** 2023-12-31

**Authors:** Putra Santoso, Arin Saparima Simatupang, Annisha Fajria, Resti Rahayu, Robby Jannatan

**Affiliations:** Biology Department, Faculty of Mathematics and Natural Sciences, Andalas University, Padang, Indonesia

**Keywords:** Alkaloids, diabetes mellitus, herbal remedies, diabetic neuropathy, malondialdehyde

## Abstract

**Objective:**

Andaliman (*Zanthoxylum acanthopodium*) is a potent medicinal plant in Asia. This present study aimed to reveal the effectivity of Andaliman fruit extract in alleviating hyperglycemia, sensory and motoric balance disorders, histopathology of the cerebellum, and tissue oxidative stress in diabetic mice induced by alloxan.

**Materials and Methods:**

Diabetes induction was performed by intraperitoneally injecting alloxan monohydrate [200 mg/kg body weight (BW)]. Subsequently, the mice were treated daily with an ethanolic extract of Andaliman fruit (0, 150, 300, 450 mg/kg BW per oral) for 28 days, followed by measurements of blood glucose, paw sensitivity, motoric balance, histopathology of the cerebellum, and malondialdehyde (MDA) levels. Moreover, the phytochemical constituents of the extract were elucidated by liquid chromatography.

**Results:**

Higher doses of Andaliman fruit extract could significantly attenuate the elevation of random and fasting blood glucose (*p* < 0.05) and improve paw sensitivity responses (*p* < 0.05) and motoric balances (*p* < 0.05) in diabetic mice. Moreover, Andaliman fruit extract could significantly attenuate the degeneration of cerebellar Purkinje cells (*p* < 0.05) and suppress MDA levels in the blood (*p* < 0.05) while blunting the MDA in the brain tissue (*p* < 0.05). Phytochemical screening revealed 39 compounds in the Andaliman extract belonging to the groups of alkaloids (26 compounds), flavonoids (12 compounds), and terpenoids (1 compound).

**Conclusion:**

The ethanolic extract of Andaliman fruit is capable of ameliorating diabetic neuropathy, motor balance disorders, and Purkinje cell degeneration while also reducing oxidative stress in the peripheral system. Hence, Andaliman extract is a promising candidate for formulation as an herbal remedy against the detrimental outcomes of diabetes mellitus.

## Introduction

Diabetes mellitus (DM) ranks among the most serious global health issues. It is estimated that there are 500 million adults worldwide suffering from DM [1]. This accounted for roughly up to 10% of the adult population. Without any proper global preventive efforts, the number will soar up to 700 million two decades later [1]. The progression of DM is profoundly implicated in various life-threatening outcomes, including the increase of cardiovascular issues [2,3], kidney diseases [4], liver diseases [5], dysregulated immune systems [6], and substantial nervous system disorders [7].

Among the various nervous system disorders promoted by diabetes, impairments of sensory and motor functions due to diabetic neuropathy are highly prevalent [8]. Chronic hyperglycemia and subsequent oxidative stress linked to diabetes have the potential to harm the nerves in the peripheral and central nervous systems, giving rise to a condition known as diabetic neuropathy and thereby reducing sensory and motor functions [9]. It has been indicated that people with DM are highly prone to developing impaired sensory function, reduced normal sensory feedback from the limbs leading to disrupted proprioception, and subsequent disability to maintain a proper motoric balance [10]. These detrimental effects on sensory and motoric balance can result in unsteadiness, decreased agile mobility, and a decline in the overall quality of daily life [10]. Therefore, managing the detrimental outcomes of DM on the nervous system, including sensory and motoric balance, is definitely essential.

Various synthetic complementary drugs have been developed and widely used to manage diabetic neuropathy and its subsequent implications, including motor balance disorder [11]. However, while aimed at managing the symptoms and complications of such health issues, common existing drugs can come with their own set of negative side effects on the gastrointestinal system, including nausea, vomiting, diarrhea, constipation [12,13], and the cardiovascular system [14]. Moreover, it had also been reported that patients who took the medications experienced dizziness, drowsiness, or difficulty concentrating [15]. In addition, certain medications for diabetic neuropathy in the form of anticonvulsants and antidepressants could lead to weight gain, fluid retention, and changes in mood and behavior [16]. Therefore, considering the various potential negative side effects of existing synthetic drugs to manage diabetic neuropathy, it is inevitably important to explore natural-based alternative medicines, including those from plants, that are effective, affordable, and have minimal side effects.

Among diverse renowned medicinal plants to counteract diseases and their subsequent detrimental outcomes, Andaliman (*Zanthoxylum acanthopodium*, Rutaceae) has garnered attention for its various potential health benefits [17]. Native to Indonesia, particularly in the North Sumatra region, this citrusy and peppery fruit is commonly employed as a spice in traditional cuisines [18]. Several studies suggest promising health advantages associated with its consumption. The fruit exhibits antioxidant properties due to its rich content of flavonoids and phenolic compounds, which can protect the body against oxidative stress and the development of chronic diseases [19,20]. In addition, Andaliman fruit extract elicited an anti-inflammatory effect *in vitro* that may have positive implications for overall health [21]. Furthermore, Andaliman fruit extract has also been reported to function as a hepatoprotector in preeclamptic rats [22] and prevent kidney and liver degeneration caused by benzopyrene [23].

Although the potential health benefits of Andaliman fruit extract have been extensively studied, research focused on its counteractive effects against DM and neuropathy remains limited. For instance, an experimental study on diabetic rats induced by streptozotocin revealed the suppressive effect of Andaliman fruit extract on triglyceride levels. However, the study did not determine the impact of the treatment on blood glucose profiles [24]. As a result, it remains unclear whether Andaliman fruit extract can manage hyperglycemia. Another study on rats suggested an antihyperglycemic effect of Andaliman fruit extract, along with a significant reduction of Schwann cells in the peripheral nerve tissue following a 14-day treatment period [25]. However, the assessment of the neuropathy indicator was based solely on the microscopic observation of Schwann cells using low magnification. It lacked behavioral assessments, such as tests for sensory and motor balance functions and histopathological observations of brain tissue. In addition, the study was conducted over a short period of time (14 days), whereas diabetic neuropathy typically develops over a longer timeframe. Consequently, a more comprehensive investigation is required to properly reveal the precise health benefits of Andaliman fruit extract in treating hyperglycemia, sensory and motor disorders, as well as the brain pathology promoted by DM. The present study was aimed at exploring these aspects. It was hypothesized that oral administration of Andaliman fruit extract could alleviate the detrimental effects of DM on hyperglycemia, sensory and motor balance, as well as brain histopathology.

## Materials and Methods

### Ethical approval

The animal handling and experimental treatments of this research were fully approved by the Board of Research Ethics and Conduct of Andalas University (KEP/12/23).

### Sample collection and extraction

The ripe fruits of Andaliman were freshly collected from Sileu-Leu, District of Sumbul, Dairi Regency, Sidikalang, North Sumatra. The species was taxonomically identified by a botanist in the Herbarium of Biology at Andalas University. Before extraction, 6 kg of Andaliman fruits were washed with tap water three times and distilled water three times before being dried at room temperature (25°C–27°C) for 3 days. Thereafter, the samples were grounded to achieve a fine structure and subsequently macerated in 70% ethanol (Sigma Aldrich). Then, the samples were stirred once every 24 h and kept in a dark place for 2 days before being filtered and concentrated using a rotary evaporator (IKA RV 8 V–RV 10 Digital V; IKA Lab, China) at 56°C. The extract was eventually stored in a dark, sealed bottle at 4°C before being used in the experiment.

### Animal conditioning and experimental treatments

#### Animal provision

The adult male Bagg Albino (BALB/c) strain mice [25 individuals; 2 months old; 23–25 gm of body weight (BW)] were provided by the Baso Veterinary Center, Bukittinggi, West Sumatra. The mice were acclimatized for a week in an animal house before being used in the experiments. The animal house was maintained at a controlled temperature (25°C–26.5°C), humidity (67%–68%), and regular light and dark cycles (12 h of light phase and 12 h of dark phase). During the acclimatization and treatment periods, mice were fed with a standard chow diet (RATBIO, CitraInna Feed, Jakarta) and tap water ad libitum.

#### Diabetic conditioning

To induce DM, 20 individuals of mice were injected with alloxan (Sigma; 200 mg/kg of BW; intraperitoneally) after an overnight fasting condition. Immediately after injection, mice were fed with a 5% sucrose solution via water bottles overnight to prevent death caused by acute hypoglycemia. After 4 days of injection, blood glucose levels were monitored using an automated glucometer (DrGlucose Almedicus) by sampling blood drops from the tail vein. The mice with a level of blood glucose ≥250 mg/dl were justified as having DM. In this present study, all 20 alloxan-injected mice met the criteria for DM. Therefore, they were eligible to be used in the experiments.

#### Experimental treatment

For the experimental purpose, mice were assigned equally into five different groups (*n =* 5) as follows:

- G1: Nondiabetic mice (non-DM; control).
- G2: Diabetic mice (DM).
- G3: Diabetic mice treated with Andaliman fruit extract 150 mg/kg BW (DM + Ext 150).
- G4: Diabetic mice treated with Andaliman fruit extract 300 mg/kg BW (DM + Ext 300).
- G5: Diabetic mice treated with Andaliman fruit extract 450 mg/kg BW (DM + Ext 450).

The extract was given orally on a daily basis (09.00–10.00 am) for 28 days. The dose levels of Andaliman fruit extract were determined based on a previous report [26], and the duration of treatments was decided in accordance with a prior study [27].

### Blood glucose and body mass measurements

At the end of the experiment, the levels of blood glucose were determined. Nonfasting blood glucose levels were examined in the morning (09.00), while fasting blood glucose levels were examined at the end of 18-h fasting (foods were removed while drinking water was available along the way). The blood drop was sampled from the vein at the tip of the tail and loaded into an automatic glucometer to determine the blood glucose values. In addition, the BW of mice was also determined using a digital balance for animals at the beginning and end of the experiment (Ohaus NV 222).

### Assessment of paw sensitivity using a hot plate test

To evaluate the sensory function of the mice (assessment of diabetic neuropathy indicator), paw sensitivity level was examined using a hot plate following the procedure as previously described [27]. Briefly, at the end of treatment, each mouse was tested by placing its left hindlimb on a hot plate at a constant temperature (50°C). The latency time of mouse responses to the hot sensation (jumping, escaping, and paw licking) was recorded using a stopwatch timer.

### Assessment of motoric balance using a rotarod and balance beam

To assess the motoric balance function, mice were tested using a balance beam and rotarod tests. The test of the balance beam was conducted as per the procedure described elsewhere [28]. In brief, each mouse was placed on a balance beam (40 cm in length), and the duration spent by mice walking on the beam from end to end was recorded. Furthermore, the rotarod test was carried out by following the procedure as previously described [29]. Before the test, mice were trained for 3 days, during which they were placed on a spinning rotarod for 5 min followed by a short break (1 min). This cycle of the pretest was repeated three times. On the day of the test, each mouse was tested by placing it on the spinning rotarod. The frequency of falling from the rotarod during the 5 min of the test was recorded using a digital camera equipped with a timer (Olympus Tough TG6 Digital Camera).

### Measurement of malondialdehyde (MDA) level

The concentrations of MDA in the blood and the brain tissue were examined by the MDA assay kit (Abcam). The procedures for measurements were carried out as per manufacturer instructions. Briefly, after the completion of all other examinations, the mice were first terminated by means of cervical vertebral dislocation. Immediately, the blood was collected, and 0.3 gm of brain tissue was sampled. Subsequently, the brain tissue was homogenized in the phosphate-buffered saline, followed by the incubation of the homogenate in a solution composed of 0.37% thiobarbituric acid in 50 nM NaOH and 2.8% trichloroacetic acid for 20 min in the shaken water bath. Furthermore, the sample was centrifuged at a speed of 1,500 rpm (Thermo Fisher Scientific) for 10 min followed by the collection of the supernatant. Eventually, the absorbance value of each sample was determined at 532 nm using a spectrophotometer (UV-Vis Biorad). For the MDA measurement in the blood plasma, the whole blood was centrifuged at 11,000 rpm for 10 min and the plasma was taken using a micropipette (Eppendorf) and processed for the MDA level measurement following the same procedures as previously performed for brain tissue homogenate.

### Histopathological examination of the cerebellum

The cerebellum samples were immediately washed with physiological saline before being soaked in buffered neutral formalin 10% overnight. Thereafter, samples were processed for histological slide preparation using graded alcohol (dehydrator) and xylene (clearing agent). The tissues were then impregnated in a block of paraffin before being cut with a microtome (Leica). The tissue slices were subjected to hematoxylin-eosin (HE) staining and sealed with cover glass glued with entelan as a mounting medium. Next, the samples were investigated using a microscope (Olympus CX 33). For each individual mouse, five different slides were examined with five different view fields. The numbers of degenerated Purkinje cells of the cerebellum were counted with ImageJ (software was freely provided by the National Institutes of Health, NIH, USA).

### Phytochemical screening of the extract

The phytochemical constituents of Andaliman fruit ethanolic extract were elucidated by using ultra-performance liquid chromatography (UPLC) coupled with quadrupole time-of-flight mass spectrometry (QTOF-MS/MS) (Xevo G2S QTOF-Waters) with particular targets, namely alkaloids, flavonoids, and terpenoids. Two grams of the extract were mixed with ethanol and ultrasonicated for 30 min followed by brief centrifugation. Moreover, the extracted sample was subjected to filtration using a membrane (GHP /PTFE mesh size of 0.22 µm) before being applied to the machine apparatus. Thereafter, 10 µl of filtered sample was loaded into the column (0.6 ml/min of flow rate). The elucidation was conducted using a chromatography column (C18 Acquity UPLC HSS T3 100 Armstrong UPLC I-Class System) with formic acid and acetonitrile as the mobile phases. The temperature of the autosampler was set at 15°C while the temperature of the column was set at 40°C. The detected compounds were determined and verified using the library UNIFI Scientific Information System that was integrated with the UPLC-QTOF-MS/MS instrument [30].

### Statistical analysis

The quantitative data were statistically analyzed for homogeneity, distribution, and variance. Subsequently, a *post hoc* test, namely Duncan’s new multiple range test, was performed (*p* < 0.05). Statistical Package for the Social Sciences version 23 (IBM) was used for the analysis.

## Results

### Effect of Andaliman fruit ethanolic extract on blood glucose and BW

**Figure 1.**
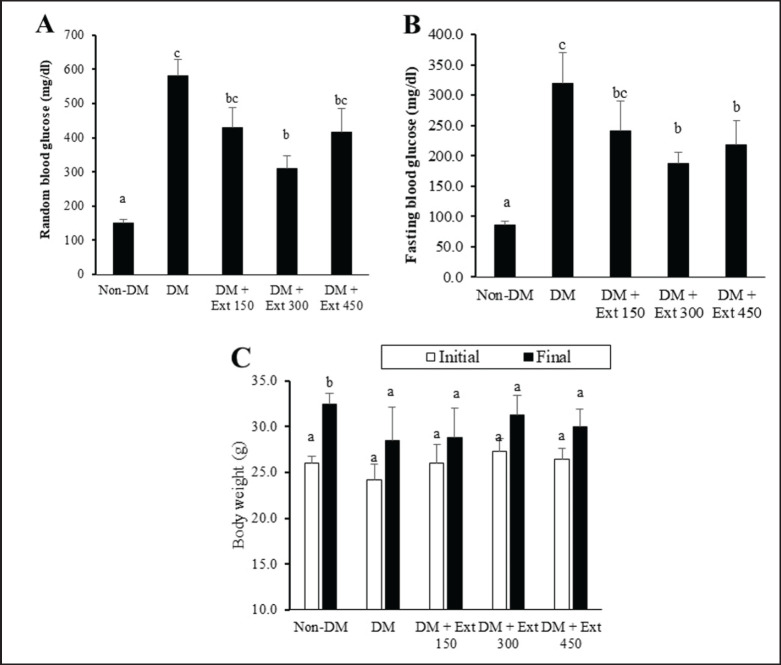
Effect of Andaliman fruit ethanolic extract on the levels of blood glucose and BW changes of mice. (A) The levels of nonfasting blood glucose, (B) fasting blood glucose, and (C) BW of mice. Non-DM (nondiabetic; healthy mice), DM (diabetic mice), Ext (extract), and lowercase letters depicted above each bar show statistical differences (*p* < 0.05).

Monitoring of random blood glucose levels (Fig. 1A) revealed that after 28 days of experimental treatments, the diabetic mice (DM group) exhibited hyperglycemia with blood glucose levels of 600 mg/dl, while the healthy mice (non-DM group) sustained a normoglycemic state (<250 mg/dl). Statistically, the blood glucose values of the two groups were significantly different (*p* < 0.01). Administration of Andaliman fruit extract showed a tendency to reduce random blood glucose levels. However, as compared with the DM group, only mice in the group treated with the extract at a dose of 300 mg/kg BW exhibited a substantial reduction in blood glucose (*p* < 0.05), while those given the other doses (150 and 450 mg/kg BW) remained statistically comparable (*p* > 0.05). Furthermore, the fasting blood glucose levels (Fig. 1B) indicated that the DM group had higher fasting blood glucose (>300 mg/dl) as compared to the non-DM group (97 mg/dl; *p* < 0.01). Oral administrations of Andaliman fruit extract, particularly at higher doses (300 and 450 mg/kg BW), could significantly reduce fasting blood glucose levels compared to the DM mice without any treatments (*p* < 0.05). However, the levels of fasting blood glucose in these groups were also markedly higher than in the non-DM group (*p* < 0.05).

In addition, measurements of BW (Fig. 1C) showed that only the non-DM group exhibited a marked gain in BW after 28 days of treatment (*p* < 0.05). In contrast, the DM group and the groups treated with Andaliman fruit extract had smaller BW changes without any substantial differences (*p* > 0.05).

### Effect of Andaliman fruit ethanolic extract on paw sensitivity

**Figure 2.**
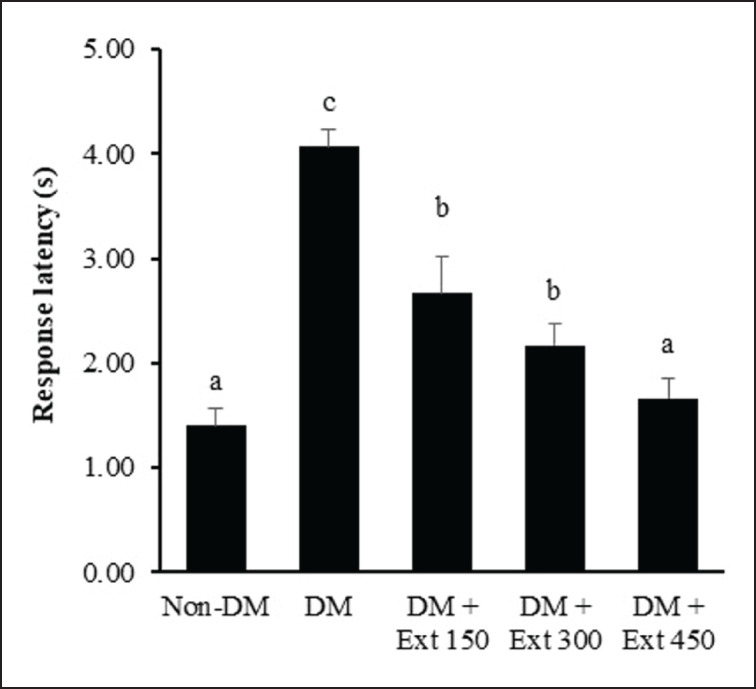
Effect of Andaliman fruit ethanolic extract on response latency of paw against hot plate in mice. Lowercase letters depicted above each bar show statistical differences (*p* < 0.05).

To assess the functional sensory response of the mice, the hot plate test was conducted. As depicted in Figure 2, the response latency of the mice in the diabetic group (DM) was markedly delayed compared to healthy mice (non-DM) (*p* < 0.01). However, mice treated with Andaliman fruit extract at all dose levels showed a reduction in latency, and these reductions were statistically significant (*p* < 0.05). The magnitude of latency reduction exhibited a dose-dependent manner, indicating that mice treated with the extract at the highest dose (450 mg/kg BW) showed a comparable response latency as nondiabetic mice (*p* > 0.05).

### Effect of Andaliman fruit ethanolic extract on motoric balance

**Figure 3.**
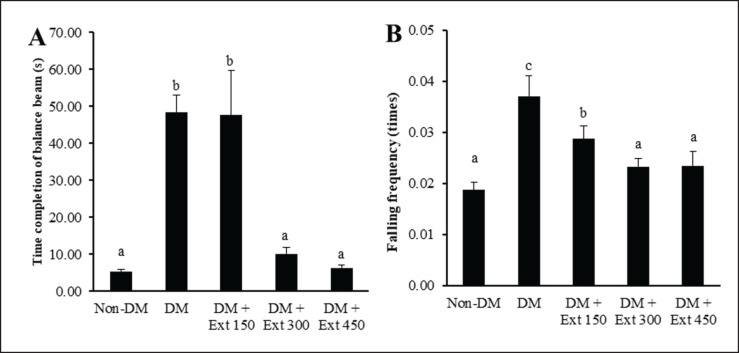
Effect of Andaliman fruit extract on motoric balance in mice. (A) Time needed to cross the balance beam and (B) frequency of falling from the rotarod. Lowercase letters depicted above each bar show statistical differences (*p* < 0.05).

The motor balance of the mice was evaluated using balance beam and rotarod tests. Data in Figure 3A showed that mice in the DM group displayed motor balance disorders, evidenced by a noticeably longer time needed to cross the balance beam than the nondiabetic group (*p* < 0.01). Similar motor balance disorders were observed in mice treated with a lower dose of Andaliman fruit extract (150 mg/kg BW). In contrast, mice receiving extract at higher doses (300 and 450 mg/kg BW) exhibited improved motor balance and even became statistically comparable to the nondiabetic mice (*p* > 0.05).

The results of the rotarod test (Fig. 3B) also indicated that DM mice had a higher frequency of falling from the spinning rotarod than nondiabetic mice (*p* < 0.01). Conversely, mice treated with Andaliman fruit extract exhibited improved motor balance, as evidenced by a significantly lower falling frequency than DM mice (*p* < 0.05). The magnitude of improvement was greater in mice treated with higher doses of Andaliman fruit (300 and 450 mg/kg BW) than in those treated with a lower dose of the extract (150 mg/kg BW).

### Effect of Andaliman fruit ethanolic extract on cerebellum histology and oxidative stress

**Figure 4.**
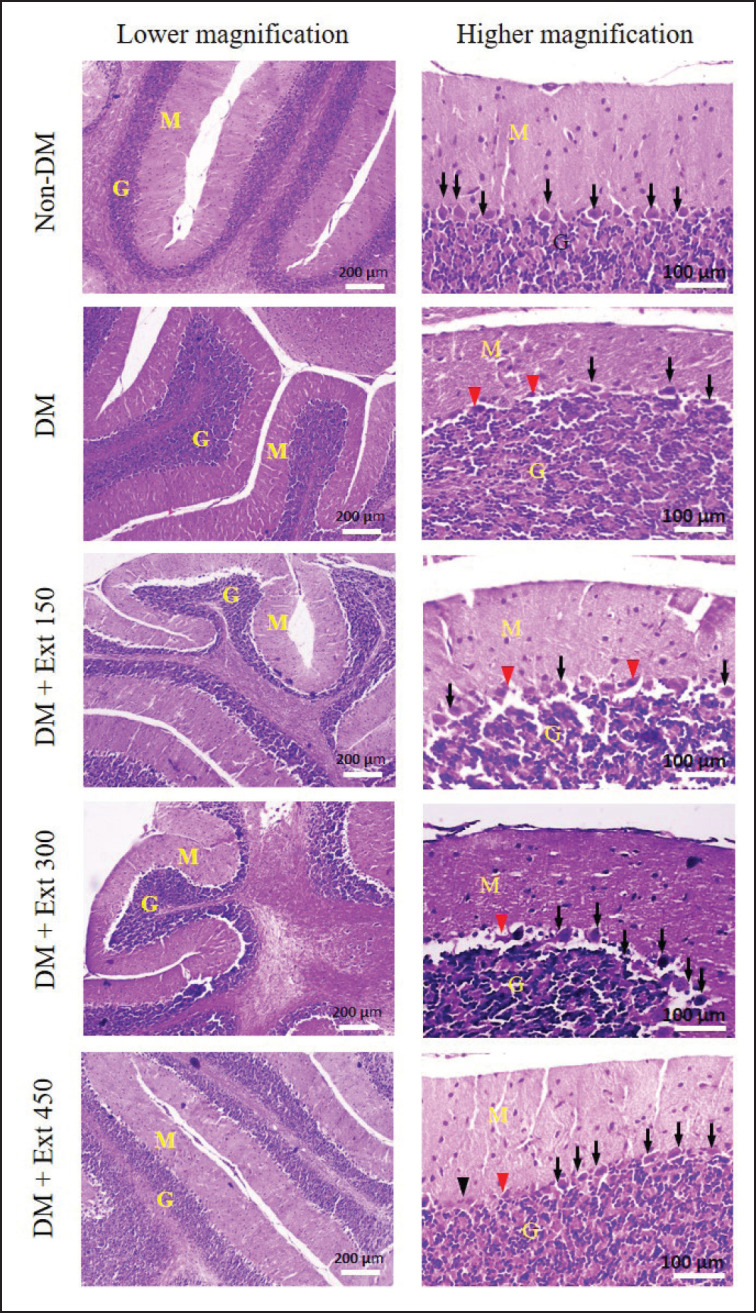
Effect of Andaliman fruit extract on histology of cerebellum in mice. M (molecular layer), G (Granular layer), black arrows (normal Purkinje cells), and red arrows (degenerated Purkinje cells). Tissues were stained with HE.

**Figure 5.**
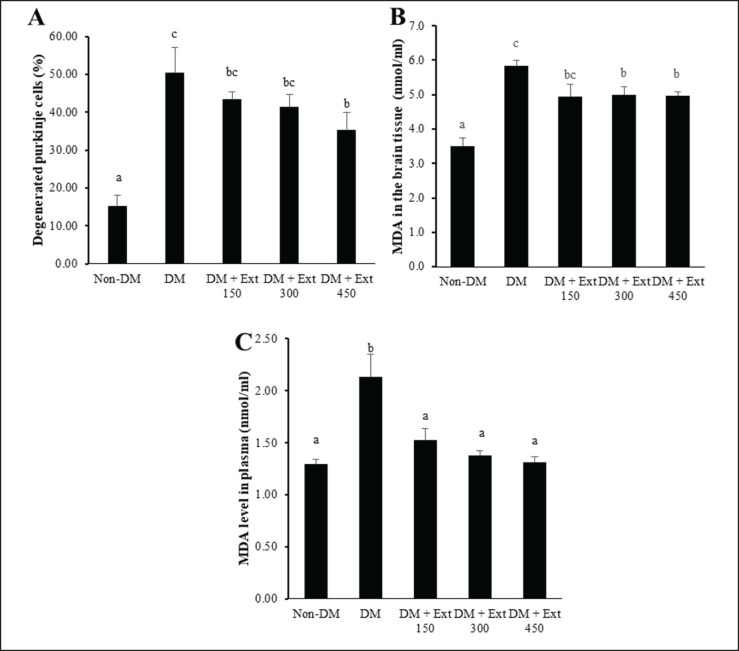
Effect of extract of Andaliman fruit on cerebellar Purkinje cells and MDA concentration in the brain tissue and blood plasma of mice. (A) Percentage of degenerated Purkinje cells in the cerebellum, (B) MDA concentration in the cerebellar tissue, and (C) MDA concentration in the blood plasma. Lowercase letters depicted above each bar show statistical differences (*p* < 0.05).

A microscopic examination of the cerebellum tissue (Fig. 4) revealed that mice in the DM group exhibited a thinner molecular layer and a reduced population of Purkinje cells. In addition, many Purkinje cells showed degeneration with lytic nuclei. In contrast, mice treated with Andaliman fruit extract displayed less atrophy of the molecular layer and predominantly normal Purkinje cells. The degeneration of Purkinje cells (Fig. 5A) significantly increased in diabetic mice (DM) as compared with nondiabetic mice (*p* < 0.01). Furthermore, mice treated with Andaliman extract also showed Purkinje cells with a lower magnitude of degeneration than mice in the DM group. However, among all the extract doses, only the highest dose (450 mg/kg BW) could significantly reduce the degeneration of Purkinje cells (*p* < 0.05). Unfortunately, the degeneration of Purkinje cells in the DM mice treated with the highest dose of Andaliman extract remained substantially different than that of the non-DM group (*p* < 0.05).

The measurement of MDA levels as a marker of oxidative stress in the brain tissue (Fig. 5B) indicated that mice in the DM group had the highest MDA level, which was statistically different from that in the non-DM group (*p* < 0.05). Furthermore, mice treated with Andaliman extract at higher dose levels (300 and 450 mg/kg BW) exhibited significantly lower MDA levels than the DM group (*p* < 0.05). However, the brain tissue’s MDA levels in the Andaliman extract-treated groups remained statistically different from those of the non-diabetic mice (*p* < 0.05). In addition, the MDA level in the blood plasma (Fig. 5C) of diabetic mice (DM) was also statistically higher than that of nondiabetic mice. In contrast, mice treated with Andaliman fruit extract at all doses had significantly lower MDA levels than the diabetic group (DM) (*p* < 0.05). Importantly, the plasma MDA levels in mice treated with Andaliman fruit extract were statistically comparable to those in the nondiabetic group (*p* > 0.05).

### Phytochemical constituents of Andaliman fruit ethanolic extract

As shown in Table 1, the phytochemical analysis found that the extract was composed of 39 compounds belonging to alkaloids (26 compounds), flavonoids (12 compounds), and terpenoids (1 compound). The majority of their potential bioactivities are as neuroprotectors or neuromodulators (14 compounds), antioxidants (9 compounds), and anti-inflammatories (16 compounds). Some of them are also known as anti-diabetes, anti-Alzheimer’s disease, anti-hypertension, and anti-aging. However, nine compounds remain unknown for their relevant bioactivity related to the scope of our present study (DM, neurological disorders, and oxidative stress).

## Discussion

The present experimental study demonstrated the beneficial effects of Andaliman fruit ethanolic extract against hyperglycemia, sensory and motoric balance disorders, histopathological alterations in the cerebellum, and MDA accumulation in diabetic mice. It was found that daily oral administration of Andaliman extract for 28 days attenuated blood glucose elevation under diabetic conditions. Andaliman extract could also substantially improve sensory and motoric balance while ameliorating the number of degenerated Purkinje cells in the cerebellum. Moreover, the extract exerted a profoundly reducing effect on MDA levels in the blood plasma while blunting MDA increases in the brain tissue. In addition, there were 39 compounds detected in the Andaliman fruit ethanolic extract belonging to alkaloids, flavonoids, and terpenoids with various relevant potential bioactivities, including anti-inflammatories, anti-oxidants, neuroprotectors, or neuromodulators.

This study indicated that the administration of Andaliman extract for 28 days failed to perfectly improve the blood glucose profile and BW to be comparable with nondiabetic (healthy) individuals. Although random and fasting blood glucose levels in Andaliman-treated mice were substantially lower than those of the DM group, their ranges remained in the hyperglycemic state (>250 mg/dl). However, a previous study in diabetic rats reported that the n-hexane extract of Andaliman fruit could substantially manage blood glucose profiles to be as good as those of healthy individuals [25]. The discrepancy shown by our study may be due to certain reasons. First, the Andaliman fruit extract may require a longer period of treatment to effectively alleviate pancreatic damage, and thereby, improving the blood glucose profile. Hence, a 28-day administration was not enough to accommodate the positive effect of Andaliman in completely alleviating pancreatic tissue degeneration against alloxan. Second, the phytochemical compounds of Andaliman fruit extracted with ethanol, as per our study, might be different from those extracted with n-hexane [31]. Consequently, the constituents and effectiveness of compounds in both extract types in managing blood glucose homeostasis could also be different. This is also supported by our phytochemical data showing that among the 39 detected compounds, only three are related to anti-diabetic activity (namely dehydroevodiamine, cytisine, and leucopelargonidin).

In line with unmanageable hyperglycemia, it appears that the administration of Andaliman fruit extract was not effective in improving the BW profile in diabetic mice. The decrease in BW is common in type 1 diabetes and could be related to several factors. First, under the conditions of insulin deficiency in type 1 diabetes, the breakdown of body fat and protein from muscle tissue is progressively elevated to compensate for the limited usable energy resources from sugar [32]. This might subsequently lead to weight loss. Furthermore, hyperglycemia could also promote increased energy expenditure to counteract impaired physiological homeostasis [33]. As a consequence, body mass is also reduced. In addition, reduced food intake and excessive urine output due to hyperglycemia in DM could also contribute to substantial weight loss. Hence, the lack of effectiveness of Andaliman fruit extract in improving the BW profile might be attributed to its inability to adequately manage hyperglycemia.

A hyperglycemic state in individuals suffering from diabetes is implicated in the increment of oxidative stress [34]. Likewise, our present findings also indicated that elevation of blood glucose led to MDA elevation as a marker of oxidative stress, while the administration of Andaliman fruit extract significantly reduced it. Chronic hyperglycemia has been linked to enhanced production of reactive oxygen species (ROS), a hallmark of oxidative stress, through various pathways. These include glucose autooxidation and the activation of enzymes such as nicotinamide adenine dinucleotide phosphate hydrogen (NADPH) oxidase [34,35]. Furthermore, hyperglycemia can also impair mitochondrial function, thereby promoting oxidative stress [35]. In addition, the generation of molecules, namely advanced glycation end products (AGEs), and the impaired functional role of endogenous antioxidants (such as catalase, glutathione peroxidase, and superoxide dismutase) due to hyperglycemia might also contribute to elevated oxidative stress in diabetes [36]. Our recent finding suggests that Andaliman fruit extract could suppress MDA levels in the blood plasma and blunt them in the brain tissue, indicating its noticeable antioxidative effect. The counteractive effect of the extract against oxidative stress could be due to some bioactive compounds contained in the Andaliman fruit. As found by phytochemical screening, nine compounds might function as antioxidants in the groups of alkaloids, flavonoids, and terpenoids. Andaliman fruit extract also highly contains carveol (a monoterpene) and linoleic acid (a fatty acid), which have a strong antioxidative effect [37,38]. However, our study found that the suppressive effect of Andaliman extract on oxidative stress in brain tissue was limited to a mild level. It is speculated that this discrepancy may be due to a higher lipid peroxidation activity promoted by hyperglycemia in the brain tissue as compared with peripheral tissues. In addition, some bioactive compounds that function as antioxidants in the Andaliman fruit extract may fail to properly penetrate the blood–brain barrier to reach the brain tissues. As a result, the Andaliman fruit extract failed to completely counteract oxidative stress in the brain despite successfully diminishing it in peripheral tissues. Further study addressing this discrepancy is needed to clarify the speculation.

Our study also revealed that the elevation of oxidative stress markers in diabetic mice was in accordance with the impairment of sensory function and motoric balance. In contrast, oral administration of Andaliman fruit extract, particularly at higher doses, could improve sensory function and motoric balance to a level comparable to that of nondiabetic individuals. There are several plausible mechanisms through which oxidative stress can deteriorate sensory function and motor balance. First, ROS can directly damage nerve cells through the lipid peroxidation process, thereby disrupting their functions in controlling sensory and motor systems [39]. In addition, oxidative stress can trigger damage in blood vessels, particularly microcapillaries, that function to supply sufficient nutrients and oxygen to the nerves [40]. As a result, the nerves could progressively degenerate, thereby impairing sensory and motor functions. Furthermore, oxidative stress can induce excessive accumulation of AGEs in nerve tissues, which in turn promotes neuronal damage, including those neurons that control sensory and motor functions [40]. Accordingly, the reduction of oxidative stress might be one of the mechanisms by which Andaliman fruit extract counteracts the detrimental effects of hyperglycemia on sensory and motoric balance.

**Table 1.** Phytochemical compounds in the group of alkaloids, flavonoids, and terpenoids detected in the ethanolic extract of Andaliman fruit.

No.	Compound name	Group	Relevant bioactivity
1	Adenine	Alkaloids	Anti-inflammatories
2	Armepavine	Alkaloids	Anti-inflammatories
3	Bufotenine	Alkaloids	Neuromodulators
4	Candicine	Alkaloids	Neuromodulators
5	Chelerythrine	Alkaloids	Unknown
6	Corybulbine	Alkaloids	Anti-inflammatories, neuroprotectors, analgesics
7	Cryptopine	Alkaloids	Neuromodulators
8	Cytisine	Alkaloids	Neuromodulators
9	Dehydroevodiamine	Alkaloids	Anti-diabetes, anti-neurodegenerative diseases, anti-inflammatories, antioxidants
10	Dihydrorutaecarpine	Alkaloids	Neuromodulators
11	d-Norpseudoephedrine	Alkaloids	Neuromodulators (stimulant), appetite suppressants
12	Gentiatibetine	Alkaloids	Anti-convulsants, neuroprotectors
13	Kokusaginine	Alkaloids	Unknown
14	Lycopodine	Alkaloids	Anti Alzheimer’s disease
15	Menisperine	Alkaloids	Anti-inflammatories
16	N,N-Dimethyl-5-methoxytryptamine	Alkaloids	Antidepression and anxiety
17	Nigeglapine	Alkaloids	Antihypertension
18	Palmatine	Alkaloids	Antioxidants, anti-inflammatories, neuroprotectors
19	Piperolactam-C5:1(2E)	Alkaloids	Unknown
20	Polycanthisine	Alkaloids	Unknown
21	Sinomenine	Alkaloids	Anti-inflammatories
22	1-[(2E,4E)-2,4-Decadie-noyl] pyrrolidine	Alkaloids	Unknown
23	2,6-Dimethyl quinoline	Alkaloids	Unknown
24	6-Hydroxydendrobine	Alkaloids	Anti-inflammatories
25	6-Methoxyisodictamnine	Alkaloids	Unknown
26	7β-Hydroxyrutaecarpine	Alkaloids	Unknown
27	Dehydrosilybin	Flavonoids	Antioxidants
28	Hispidulin	Flavonoids	Antioxidants, neuroprotectors, anti-epileptic, anti-inflammatories
29	Isorhamnetin-3-O-neohesperidoside	Flavonoids	Anti-inflammatories
30	Kaempferol-3-glucuronide	Flavonoids	Anti-inflammatories
31	Kaempferol-3- O-β- D-glucopyranoside	Flavonoids	Antioxidants, anti-inflammatories
32	Leucopelargonidin	Flavonoids	Antidiabetes
33	Morin	Flavonoids	Antioxidants, anti-diabetic, anti-inflammatories, antihypertension, neuroprotectors
34	Mururin A	Flavonoids	Unknown
35	Nelumboroside A	Flavonoids	Antioxidants
36	Oroxin B	Flavonoids	Anti-inflammatories
37	Procyanidin B7	Flavonoids	Anti-inflammatories
38	Rhamnetin	Flavonoids	Antioxidants, anti-inflammatories
39	Retinol	Terpenoids	Antioxidants, anti-aging

Purkinje cells, located in the cerebellar cortex, have a critical role in motor control, particularly in the coordination and planning of movements [41]. Damage to these cells is implicated in the disruption of motor balance [42]. It has been shown that hyperglycemic exposure significantly causes the degeneration of Purkinje cells [42]. Another report demonstrated that hyperglycemia in diabetic animals promoted degeneration through mechanisms involving mitochondrial damage and increased release of ROS in neurons [43]. Similarly, our current study found that the hyperglycemic state in diabetic mice noticeably promoted Purkinje cell degeneration. Otherwise, the administration of Andaliman fruit extract elicited a mild counteractive effect against it. The reduction of Purkinje cells by the Andaliman extract could be due to its inhibitory effect against ROS production and mitochondrial damage. Unfortunately, the preventive effect of Andaliman fruit extract against mitochondrial damage was not determined in this study. However, the data regarding MDA levels in both the brain and blood plasma support the speculation that the protective effect of Andaliman fruit extract on Purkinje cells might be mediated via the inhibition of ROS production. Interestingly, despite the lesser effectiveness of Andaliman fruit extract in counteracting Purkinje cell degeneration, the mice treated with Andaliman fruit extract exhibited better motoric balance performance. There is a possibility that the survived Purkinje cells were sufficient to accommodate the normal state of motoric balance. As a result, the performance of motoric balance was well sustained despite a substantial decrease in Purkinje cell numbers. The phytochemical screening found various substances in the Andaliman fruit extract that could function as neuroprotectors or neuromodulators (14 compounds), thereby eliciting a protective effect on neurons, including Purkinje cells in the cerebellum. However, it is also speculated that the concentrations of the bioactive compounds that function as neuroprotectors or neuromodulators in the Andaliman fruit extract might not be adequate to elicit a fully protective effect on the Purkinje cells. Alternatively, the compounds could also not maximally penetrate the blood–brain barrier, thereby failing to act on the brain tissues. As a result, the number of Purkinje death cells due to hyperglycemia remained higher in those treated with Andaliman fruit extract. Further investigation is needed to confirm this speculation.

In addition to oxidative stress, inflammatory responses are also profoundly implicated in promoting the pathophysiology of neuropathy in diabetic patients [44]. Mechanistically, the inhibition of inflammatory responses is indicated to effectively alleviate neuropathy [45]. In this present study, at least 16 compounds were detected in the ethanolic extract of Andaliman fruit that might function as anti-inflammatory agents. Thus, the beneficial effects of Andaliman fruit against neuropathy, which leads to sensory and motoric balance disorders, could also be mediated by bioactive compounds in the extract that function to manage inflammatory responses. An experimental study conducted on streptozotocin-induced diabetic rats revealed a close association between the elevation of inflammatory cytokines in brain tissues, such as interleukin-6 and tumor necrosis factor alpha, and the initiation and progression of neuropathy [39]. Furthermore, the administration of an extract rich in anti-inflammatory compounds demonstrated marked effectiveness in mitigating inflammation and enhancing the structure of the brain, including the Purkinje cells in the cerebellum [39]. A previous study also revealed that Andaliman fruit extract is capable of suppressing inflammatory mediators, indicating its beneficial effect as an anti-inflammatory [46]. Unfortunately, in the current study, we did not determine the levels of inflammatory markers, such as proinflammatory cytokines, differential leukocyte counts, or the degree of macrophage infiltration in the tissues. Consequently, it remains unclear whether the beneficial effect of Andaliman fruit ethanolic extract against diabetic neuropathy is also facilitated by its suppressive action against diabetes-induced inflammatory responses.

Several weak points of the present study should be considered. First, this research was performed over a relatively short period of time (28 days of treatment), which hinders us from defining whether a prolonged period of treatment may elicit better health outcomes or otherwise. Moreover, this study tested a crude extract, not a purified one. As a result, it remains unknown which particular compounds in the extract play a major role in exerting beneficial effects on health. The phytochemical screening in this study targeted a limited number of compound groups (alkaloids, flavonoids, and terpenoids), leaving other groups of compounds unexplored. Next, the endogenous antioxidants such as catalase, glutathione, and superoxide dismutase were not determined. Furthermore, the histopathology of the sciatic nerve, which is common in diabetic neuropathy, was not investigated. In addition, the molecular mechanistic aspects of the Andaliman extract in overcoming the detrimental effects of DM were not revealed. Hence, future studies addressing the limitations are required to enhance our understanding of the potential of Andaliman fruit as one of the candidates for natural-based drugs.

## Conclusion

This current study demonstrated that oral administration of Andaliman fruit ethanolic extract at higher doses could attenuate hyperglycemia and improve paw sensitivity response and motoric balance in alloxan-induced diabetic mice. Moreover, Andaliman fruit extract was capable of ameliorating the degeneration of cerebellar Purkinje cells and profoundly counteracting systemic oxidative stress while preserving it in the brain tissue. These beneficial effects could be related to various bioactive compounds (39 compounds) found in the extract that function as neuroprotectors or neuromodulators, antioxidants, and anti-inflammatory agents. Hence, the Andaliman fruit extract is a promising candidate to be formulated as an herbal remedy against the detrimental outcomes of DM.

## List of abbreviations

BALB: Bagg Albino; BW: body weight; DM: diabetes mellitus; GHP/PTEF: type G hydrophilic polypropylene/polytetrafluoroethylene; HE: hematoxylin-eosin; MDA: malondialdehyde; NADPH: nicotinamide adenine dinucleotide phosphate hydrogen.
